# Internal root resorption: understanding the pink tooth of mummery and its clinical implications

**DOI:** 10.4317/jced.63932

**Published:** 2026-04-25

**Authors:** Halex de Souza Mercante, Gabriel Pereira Nunes, Priscila Toninatto Alves de Toledo, Michela Melissa Duarte Seixas Sostena, Larissa Pereira Nunes, Hugo Rodrigues Gonçalves, Tamires Passadori Martins, Ana Paula Miranda Vieira

**Affiliations:** 1 Department of Dentistry, Integrated School of Três Lagoas, Association for Education and Culture of Mato Grosso do Sul (AEMS), Três Lagoas, MS, Brazil; 2 Department of Prosthodontics and Periodontology, Piracicaba Dental School, University of Campinas (UNICAMP), Piracicaba, SP, Brazil; 3 Department of Prosthodontics and Periodontology, Piracicaba Dental School, University of Campinas (UNICAMP), Piracicaba, SP, Brazil; 4 Department of Operative Dentistry, Endodontics and Dental Materials, Bauru School of Dentistry, São Paulo University (USP) Bauru, SP, Brazil; 5 Department of Dentistry, Pontifical Catholic University of Minas Gerais (PUC-MG), Belo Horizonte, Minas Gerais, Brazil; 6 Department of Dentistry, United School of Northern Minas (Funorte), Ipatinga, Minas Gerais, Brazil6 Department of Preventive and Restorative Dentistry, São Paulo State University (UNESP), School of Dentistry, Araçatuba, SP, Brazil

## Abstract

**Background:**

Root resorption may be physiological or pathological. Physiological resorption occurs in deciduous teeth to allow replacement by permanent successors. In permanent dentition, resorption is pathological and classified as external (ERR) or internal (IRR), depending on its origin. IRR begins within the root canal and may lead to progressive dentin destruction. Although often asymptomatic, advanced cases may present as a pink discoloration of the crown, known as Pink Tooth of Mummery (PTM). To review current scientific evidence regarding the etiology, pathogenesis, classification, diagnosis, and treatment of IRR, as well as its clinical association with PTM.

**Material and Methods:**

This review was conducted using a structured search of PubMed, Scopus, LILACS, and SciELO. Studies addressing IRR in human teeth and aspects related to its etiology, pathogenesis, classification, diagnosis, or treatment were included. Reports involving PTM associated with internal or external resorptive processes were also considered. Data were extracted and qualitatively synthesized.

**Results:**

A total of 62 articles were selected for analysis. Trauma was the most frequently reported etiological factor, followed by orthodontic movement, pulpotomy procedures, pulp infections, and tooth autotransplantation. IRR is classified as internal surface, internal inflammatory, or internal replacement resorption, based on biological and pathological features. Diagnosis relies on clinical examination and imaging, with cone beam computed tomography improving assessment of lesion location and extent. Conventional endodontic treatment remains the primary management strategy in favorable cases. PTM is not exclusive to IRR and may also occur in external or physiological resorption. The literature on PTM consists predominantly of case reports.

**Conclusions:**

Early diagnosis is essential for the appropriate management of IRR. While bioactive materials and regenerative approaches have been explored, current evidence remains limited, particularly regarding PTM, highlighting the need for further clinical studies.

## Introduction

Root resorption is a complex biological process characterized by the loss of mineralized dental tissues due to the activity of odontoclastic cells. This phenomenon may occur under physiological or pathological conditions, leading to the degradation of dentin, cementum, or surrounding bone structures (1 , 2). The process is mediated by inflammatory and cellular mechanisms that disrupt the protective layers of the tooth, allowing clastic cells to adhere to and resorb mineralized tissues. Depending on its location and underlying cause, root resorption may present distinct clinical and radiographic features, requiring careful evaluation for accurate diagnosis. In the deciduous dentition, root resorption is considered a physiological event. The process of rhizolysis enables the exfoliation of primary teeth and their replacement by permanent successors. In contrast, in the permanent dentition, root resorption is pathological and may compromise tooth integrity and longevity. It can affect either the external root surface or the internal walls of the root canal system and is therefore classified as external root resorption (ERR) or internal root resorption (IRR), respectively (3). While ERR is more frequently reported in clinical practice, IRR remains a distinct and less common pathological entity with specific biological and clinical characteristics. The first description of IRR in a permanent lower first molar was reported by Bell in 1837 (4). Although considered relatively rare, with an estimated prevalence ranging between 0.01% and 1% (5), its true occurrence may be underestimated due to its asymptomatic and insidious progression. In many cases, IRR is detected incidentally during routine radiographic examinations or when it has already reached an advanced stage (3). When the resorptive process extends coronally, vascular granulation tissue may become visible through the enamel, resulting in a pinkish discoloration of the crown known as the Pink Tooth of Mummery (PTM), named after James Howard Mummery, who first described the phenomenon (6). Given its potential for silent progression and structural compromise, early recognition of IRR is critical to prevent extensive destruction and possible tooth loss. A comprehensive understanding of its etiology, pathogenesis, classification, clinical and radiographic characteristics, and therapeutic approaches is essential for accurate diagnosis and appropriate treatment planning. Therefore, this narrative review aimed to present and critically discuss the current scientific literature on internal root resorption, emphasizing its biological basis and clinical management.

## Materials and Methods

This study was conducted as a narrative literature review aimed at synthesizing the current scientific evidence on internal root resorption and its clinical association with the Pink Tooth of Mummery. A search strategy combining keywords and free-text terms was applied to identify relevant studies in the electronic databases PubMed, Scopus, LILACS, and SciELO. The initial search strategy was developed for PubMed and then adapted to ensure compatibility with the other databases (see Supplementary Material - Appendix A). In addition, a manual search of the reference lists was conducted to identify potentially relevant studies that might not have been captured through the electronic search. No restrictions were applied regarding publication date or language. Studies were considered eligible if they: (a) addressed internal root resorption in human teeth; (b) discussed aspects related to its etiology, pathogenesis, classification, diagnosis, or treatment; and (c) consisted of clinical studies (randomized or observational), case series, case reports, systematic reviews, or narrative reviews. (d) For the specific analysis of Pink Tooth of Mummery, studies were included when they reported clinical cases or discussions involving pink discoloration associated with internal or external resorptive processes. Studies were excluded if they: (a) were limited to animal or in vitro experiments; (b) focused exclusively on external root resorption without any association with Pink Tooth of Mummery; (c) described inflammatory internal resorption secondary to advanced pulpal necrosis with extensive periapical abscess formation; (d) lacked sufficient clinical or radiographic information to support the diagnosis; or (e) were editorials, letters, or opinion articles without primary data. Two reviewers independently conducted the study selection and data extraction processes. Duplicate records were identified and removed based on title comparison during the selection process. Data extraction and verification of the selected studies were performed by the same independent reviewers to ensure consistency (7). After applying the inclusion and exclusion criteria, a total of 62 articles were included in this narrative review.

## Etiology

The etiology of internal root resorption (IRR) has been organized by Heboyan et al. (2022) (8) into two broad categories: endogenous and exogenous factors. This classification facilitates clinical reasoning by distinguishing patient-related susceptibility from externally induced triggers. Endogenous factors are intrinsic to the individual and include age, sex, ethnicity, and genetic predisposition (9). Although these factors cannot be modified, they may influence tissue response and susceptibility to resorptive processes. Exogenous factors, in contrast, are related to local or therapeutic events and include orthodontic movement, dental trauma, pulpotomy procedures, pulp infections, and tooth autotransplantation (8). Because many of these factors are iatrogenic or trauma-related, their identification is especially relevant for prevention and early diagnosis. Systemic conditions have also been associated with IRR. Between 1986 and 2016, four publications reported cases of herpes zoster involving the trigeminal nerve in association with IRR (10), suggesting that systemic viral involvement may, in specific circumstances, contribute to pulpal alterations. Below, the main exogenous factors-particularly those with preventive potential-are discussed in greater detail. Orthodontic movement: Orthodontic forces, when properly planned and controlled, promote physiological remodeling. However, excessive or improperly distributed mechanical stress may initiate inflammatory responses within the pulp, potentially contributing to IRR (11). Therefore, orthodontic planning must consider individual biological variability (endogenous factors), while ensuring that forces are carefully calibrated and monitored throughout treatment (12). Dental trauma: Dental trauma is widely regarded as one of the principal etiological factors of IRR. De Souza et al. (2020) (13) evaluated the relationship between trauma type and IRR occurrence, reporting higher incidence rates following extrusive luxation (2.25%-13.3%), followed by subluxation (1.54%-1.72%) and lateral luxation (0.0%-1.45%). An earlier report identified IRR in 1.2% of teeth reimplanted after avulsion (13). Higher frequencies were described by Qassem et al. (2015) (14), who reported IRR in 5% of cases after subluxation and 2% after intrusive luxation. Root fractures resulting from trauma have also been associated with IRR development (15 , 16). Clinical presentation appears to influence detection and risk assessment. Cases with evident clinical signs, including fractures, discoloration, or fistulas, tend to demonstrate a higher incidence of IRR compared to cases without overt manifestations. This observation is supported by a retrospective study of 674 children who experienced traumatic events without clear clinical signs and presented only radiographic findings; among these cases, only one instance of IRR was identified (17). Tooth autotransplantation: Autotransplantation is primarily indicated for immature teeth with incomplete root formation and open apices. Although generally considered a viable therapeutic option, complications may occur. In a study evaluating 366 autotransplanted teeth, IRR was reported in three cases (1.1%), with onset predominantly between the second- and sixth year following transplantation (18). Pulpotomy: Pulpotomy aims to preserve radicular pulp vitality by removing infected coronal pulp tissue while maintaining the remaining pulp structure. Despite its conservative intent, IRR is recognized as a possible postoperative complication (19). In deciduous teeth, IRR has been described as the most frequent radiographic alteration following pulpotomies performed with zinc oxide-eugenol paste, calcium hydroxide, or formocresol as capping agents (20 , 21). Li et al. (2016) also reported a high incidence of IRR when 5% sodium hypochlorite was used as the sole medicament. Conversely, a narrative review indicated a lower incidence of IRR when ferric sulfate was used compared to calcium hydroxide (22). More favorable outcomes have been reported with mineral trioxide aggregate (MTA). In a study involving 20 patients, no cases of IRR were observed after MTA pulpotomy (23). Similarly, MTA combined with either high-power (n=21) or low-power laser therapy (n=21) showed no IRR over an 18-month follow-up period (24). When newer biomaterials were evaluated, such as 3Mixtatin, IRR was observed only in the formocresol group (2.6%) (25). In another comparison including electrocautery, MTA, and bioactive glass, formocresol again demonstrated the highest occurrence of IRR, with eight cases among 20 patients (26). In permanent teeth, the use of silicate-based cement (Biodentine, Septodont, Saint Maur-des-Fossés, France) produced favorable outcomes, with only one reported case of IRR in a sample of 20 molars (27). Pulp infections: Pulpal inflammation represents a key biological pathway in the development of IRR. Bacterial invasion may occur through dentinal tubules, carious lesions, fissures, fractures, or lateral canals (28). Once established, infection can initiate and sustain an inflammatory response capable of activating clastic cells within the pulp space, thereby contributing to the resorptive process.

## Pathogenesis and classification

**Figure 1:**
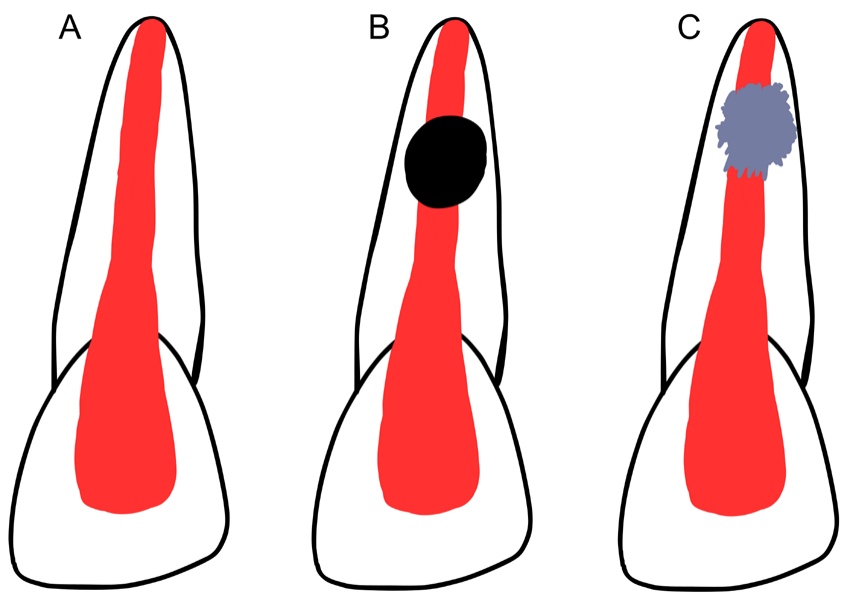
Illustration of internal root resorption. (A) Healthy tooth. (B) Tooth with internal inflammatory resorption. (C) Tooth with internal replacement resorption.

The internal surfaces of the root canal are protected by a natural anti-resorptive barrier composed of the odontoblastic layer and predentin, which line and protect the underlying dentin. Clastic cells are unable to adhere to or resorb non-mineralized tissues. For this reason, resorption is initiated only when an inducing factor disrupts this protective layer and exposes the mineralized dentin surface (28). Resorptive activity may be transient and followed by repair if the stimulus is mild and self-limited. However, when pulpal inflammation persists, a sustained clastic response may develop, resulting in progressive degradation of dental hard tissues (11 , 29 , 30). The balance between tissue injury, inflammatory response, and vascular supply plays a central role in determining whether the process will resolve or progress. Over the years, several classifications of root resorption have been proposed, based on etiology, anatomical location, and therapeutic implications (31). According to Aidos et al. (2018) (30), fifteen different classification systems were published between 1970 and 2016, including the widely referenced classification proposed by Andreasen (1994) (32). In this review, emphasis is given to the more recent classification proposed by Abbott and Lin (2022) (29), which is grounded primarily in anatomical, physiological, and pathological mechanisms involved in resorption. - Internal Surface Resorption Internal surface resorption represents a transient and self-limiting phenomenon without clinical manifestations. It may occur in two distinct situations. The first involves minor pulpal irritation in the absence of infection, with spontaneous resolution typically within two to three weeks. The second corresponds to an initial stage preceding internal inflammatory resorption, before pulpal infection and necrosis are established (29). Because it is temporary and often reversible, this form of resorption is rarely detected clinically and tends to remain unnoticed unless progression occurs - Internal Inflammatory Resorption The designation "inflammatory" reflects the presence of an inflammatory process within the root canal system. In this condition, intraradicular dentin is progressively degraded and replaced by granulation tissue. Adjacent to the resorptive area, necrotic tissue may be present; however, a blood supply is maintained through the apical foramen or through accessory canals connecting to the periodontal ligament, preserving at least partial pulp vitality (29 , 33). This vascular supply is a defining feature of internal inflammatory resorption and explains its relative rarity when compared to external root resorption. Clastic cells require nutritional support provided by vital pulp tissue to remain active (34). As the lesion progresses, two possible outcomes may occur. The resorptive defect may enlarge and eventually perforate the root canal wall, establishing communication with the periodontal tissues. Alternatively, progression may continue until complete pulpal necrosis develops (Fig. 1).

1

**Figure 2:**
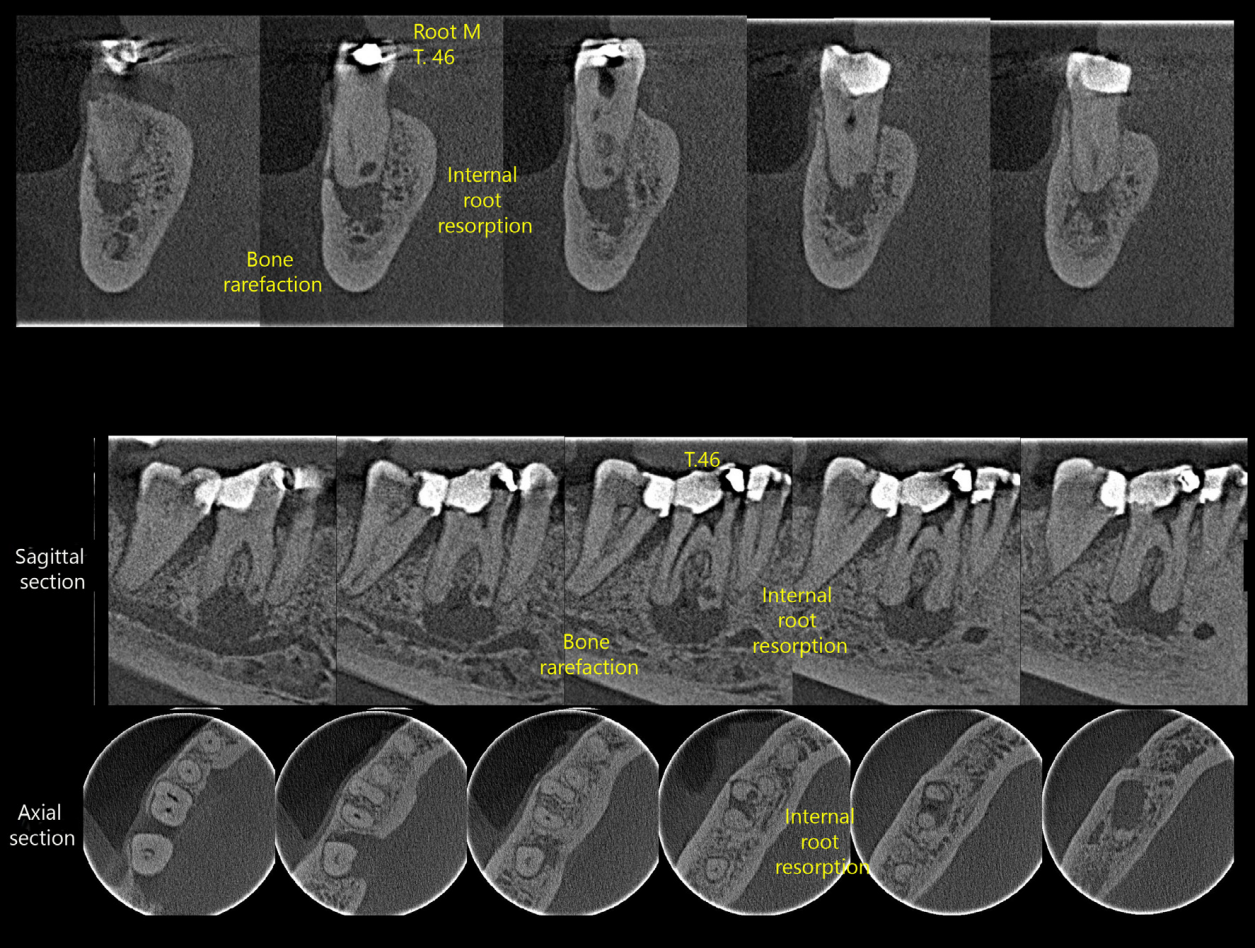
Computed tomography as an imaging test for the differential diagnosis of internal root resorption.

Once total necrosis occurs, the vascular supply ceases, clastic cells lose viability, and the resorptive process is interrupted. At this stage, apical periodontitis commonly develops as a consequence of microbial infection (35 , 36). Clinical presentation varies according to pulpal status. Patients may remain asymptomatic or may present signs and symptoms consistent with acute pulpitis or apical periodontitis. In cases of root perforation, the formation of a sinus tract is frequently observed. Radiographically, internal inflammatory resorption is characterized by a well-defined, oval or circular radiolucent enlargement of the root canal space, often described as balloon-like in appearance, with symmetrical expansion of the canal walls (2) (Fig. 2).

2

- Internal Replacement Resorption In internal replacement resorption, normal pulp tissue is gradually substituted by a combination of granulation tissue and a mineralized tissue resembling bone. This newly formed hard tissue is described as metaplastic due to its structural and biological differences from dentin (8). This form of resorption is typically asymptomatic. Pulp sensibility tests may initially produce positive responses, reflecting residual vitality, although responses tend to diminish as the lesion advances. Radiographically, internal replacement resorption appears as an irregular enlargement of the root canal space, partially filled with a material that is less radiolucent than dentin. The normal root canal outline becomes distorted, and the root contour may appear altered (2), (Fig. 1).

## Differential diagnosis

The diagnosis of internal root resorption is established through the integration of clinical examination, imaging findings, and pulp vitality testing (37). Among these components, radiographic assessment plays a decisive role, not only in identifying IRR but also in distinguishing it from external cervical resorption, a condition with a similar radiographic presentation but different biological behavior and therapeutic implications. On conventional radiographs, external cervical resorption is typically characterized by preservation of the original contour of the root canal walls. In contrast, IRR presents as a symmetrical enlargement of the canal space, with loss of the normal internal outline due to intraradicular dentin resorption (38). This distinction is fundamental for accurate diagnosis and treatment planning. To refine the differential diagnosis, Clark's radiographic technique, based on the parallax principle, may be employed. By altering the horizontal angulation of the X-ray beam, it becomes possible to evaluate the spatial relationship between the lesion and the root canal. In cases of external cervical resorption, the lesion appears to shift position according to the direction of tube angulation. Lesions located on the palatal or lingual surface move in the same direction as the tube shift, whereas those on the buccal surface move in the opposite direction. Conversely, lesions of internal origin remain centered within the canal space, maintaining their relative position despite changes in angulation (39). Although periapical radiography remains widely used in clinical practice, it is inherently limited by its two-dimensional representation of three-dimensional structures (40). Superimposition of anatomical features and the inability to assess depth may hinder precise evaluation of lesion extent and possible perforation. In this context, cone beam computed tomography has emerged as a valuable adjunctive imaging modality, especially in complex or doubtful cases. CBCT allows three-dimensional visualization of the resorptive defect, enabling more accurate assessment of its location, size, and relationship with surrounding structures (41). Greater sensitivity has been reported in detecting resorptive lesions and identifying perforations that may not be evident on conventional radiographs. However, diagnostic performance is not solely dependent on the imaging modality. Martins et al. (2021) reported higher diagnostic failure rates with CBCT compared to periapical radiography. This finding has been attributed, at least in part, to variability in professional training and experience in interpreting tomographic images. These observations underscore the importance of adequate training in CBCT analysis to ensure reliable diagnostic outcomes. The relevance of CBCT in the diagnosis, treatment planning, and follow-up of root resorption has been recognized by professional organizations such as the European Society of Endodontology, the American Association of Endodontists, and the American Academy of Oral and Maxillofacial Radiology (42). When judiciously indicated and properly interpreted, CBCT represents an important complementary tool in the differential diagnosis of internal and external resorptive lesions.

## Interventions

The management of internal root resorption begins with a careful assessment of tooth restorability. Clinical and radiographic evaluation should determine whether sufficient structural integrity remains to justify intervention. When the defect is extensive, and the tooth cannot be predictably restored, extraction followed by appropriate rehabilitation is indicated. In cases considered favorable for treatment, prognosis is largely influenced by the size and location of the lesion, as well as by the presence or absence of perforation. The primary therapeutic objective is to halt the resorptive process, which depends on complete removal of vital pulp tissue and elimination of the vascular supply sustaining clastic activity (37). The treatment approach is guided by the accessibility of the defect and the integrity of the remaining root walls. When the resorptive cavity can be reached through conventional endodontic access, nonsurgical root canal treatment is indicated. If the defect is inaccessible to mechanical instrumentation or if perforation compromises proper debridement, surgical intervention may be required. Regardless of the technique employed, effective disinfection of the root canal system using appropriate irrigants and intracanal medications remains essential (2 , 28). In an effort to enhance disinfection, alternative adjunctive methods have been investigated, including electrosurgical pulpectomy and diode laser application (43 , 44). These approaches aim to improve the decontamination of irregular resorptive areas that may be difficult to instrument mechanically. The choice of filling material depends primarily on the condition of the root wall. In teeth with intact walls, gutta-percha associated with an adequate sealer is generally recommended. In cases involving perforation, bioactive materials are preferred because of their ability to promote mineralized tissue deposition and support repair. Although calcium hydroxide has been widely described in the literature, its high solubility limits its role to that of an intracanal dressing rather than a definitive filling material (45). Materials with superior sealing ability are therefore indicated, particularly mineral trioxide aggregate and bioceramic cements (46). Recently, regenerative endodontic therapy has been proposed as a potential alternative in selected cases, especially when preservation or reestablishment of pulp vitality is desirable. This approach seeks to replace damaged dental structures with vital tissue capable of restoring biological function (8). Nageh et al. (2022) (36) reported clinical resolution of signs and symptoms of IRR in 13 necrotic incisors treated with platelet-rich fibrin. Although these findings are encouraging, clinical evidence remains limited. Additional well-designed studies are necessary to clarify the predictability and long-term effectiveness of regenerative strategies in the management of internal root resorption (8).

## Pink tooth of mummery

**Figure 3:**
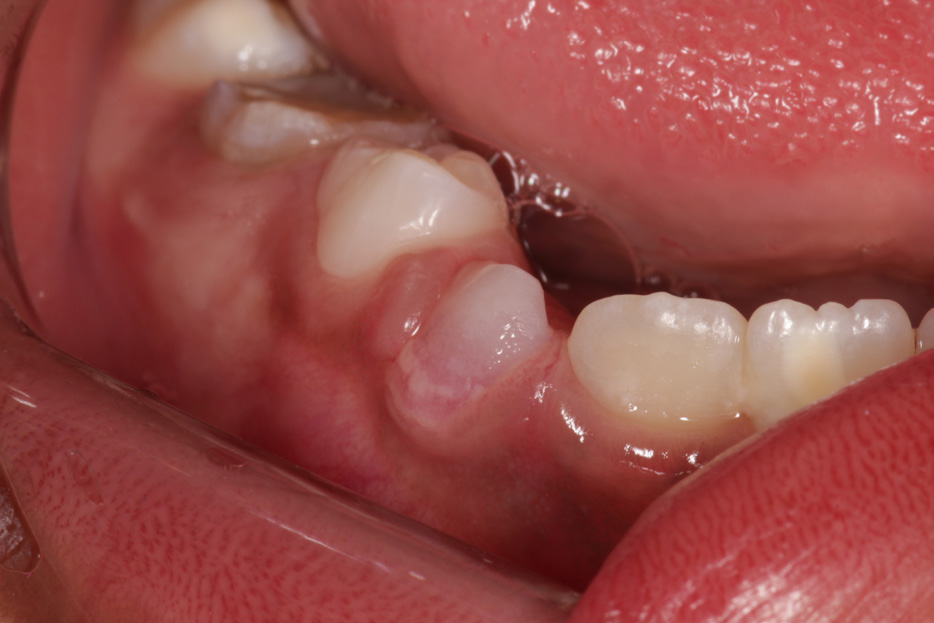
Pink tooth of Mummery with physiological etiology.

For many years, pink discoloration of the crown was considered a pathognomonic sign of internal root resorption (47). Subsequent observations demonstrated that this clinical presentation is not exclusive to IRR. The pink appearance has also been described in cases of physiological resorption and in invasive cervical resorption (Fig. 3), a specific subtype of external root resorption (48 , 49).

3

In internal root resorption, the pink hue results from inflamed and vascularized pulp tissue occupying the resorptive cavity. Because enamel is partially translucent, the underlying granulation tissue becomes visible through the crown, producing the characteristic pink stain. In invasive cervical resorption, however, the discoloration originates from highly vascular periodontal granulation tissue that proliferates externally and extends into the cervical region of the tooth (50). This clinical similarity reinforces the importance of careful diagnostic evaluation. Differentiating between internal and external resorptive processes is essential, as treatment strategies and prognosis vary considerably according to the underlying pathology (51 , 52). Reports specifically addressing Pink Tooth of Mummery remain limited in the literature, underscoring its rarity (Table 1).

Table 1

One of the earliest descriptions was provided by Marshall in 1960 (53), who documented a case affecting an anterior tooth and associated it with trauma that had occurred approximately twenty years earlier. In subsequent case reports, dental trauma has consistently emerged as the most frequently cited triggering factor, suggesting a possible relationship between prior pulpal injury and the development of the characteristic pink discoloration. Segura-Egea et al. (2011) (57) described a case involving a pink discoloration in the maxillary right central incisor that became evident two decades after a traumatic injury. Clinical and radiographic examination revealed a well-defined, oval radiolucent area located in the middle third of the root, leading to the diagnosis of internal root resorption. Among the therapeutic options presented, the patient elected to undergo conventional endodontic treatment. Chemomechanical preparation was performed using 5.25% sodium hypochlorite as the irrigant, followed by placement of a calcium hydroxide intracanal dressing maintained for thirty days. The canal was subsequently obturated with gutta-percha and gray mineral trioxide aggregate, and the tooth was restored. After 24 months of follow-up, the outcome was considered clinically successful. Nevertheless, a greenish discoloration of the crown was observed, which the authors attributed to the use of gray MTA. This case emphasizes that, especially in coronal resorptive defects, the choice of biomaterial must take into account not only biological compatibility and sealing ability but also the potential for tooth discoloration. Deep et al. (2021) (47) reported Pink Tooth of Mummery following replantation of an avulsed tooth. The patient, a 12-year-old girl, had experienced avulsion of the left maxillary central incisor at eight years of age. Clinical examination revealed a pink stain and grade II mobility. Radiographic evaluation demonstrated extensive resorption affecting the buccal aspect of the root. Because implant placement was contraindicated due to the patient's age and ongoing skeletal development, a surgical approach was chosen despite the unfavorable long-term prognosis. The resorptive area was surgically accessed, and 90% aqueous trichloroacetic acid was applied for one to two minutes. This was followed by curettage, canal instrumentation, obturation with gutta-percha and white MTA, and restorative sealing. The authors acknowledged that extraction would ultimately be inevitable given the extent of the lesion. However, the treatment was performed with the intention of preserving the natural tooth and maintaining alveolar bone volume until skeletal maturity was reached. A similar rationale was presented by Muñoz-Sánchez et al. (2021) (64), who described a case managed with a conservative, temporizing objective. A 10-year-old patient had suffered intrusive luxation of the maxillary lateral incisors. Endodontic treatment was initiated three years after the traumatic event, and two years later pink discoloration became apparent. The diagnosis was invasive cervical resorption. Management involved surgical exposure of the root surface, instrumentation, irrigation with sodium hypochlorite, obturation with gutta-percha and tricalcium silicate-based material, and restorative sealing. Despite these measures, both teeth were eventually avulsed one year later. This report highlights that, in pediatric patients with extensive resorption, treatment decisions must consider age and developmental stage. Even when prognosis is limited, minimally invasive or interim approaches may be justified to preserve dental and alveolar structures for as long as possible. Rodrigues et al. (2021) (65) reported a case of pink discoloration associated with previous dental trauma. Disinfection of the root canal system was performed using sodium hypochlorite with ultrasonic agitation, followed by mechanical instrumentation and placement of calcium hydroxide for ten days. The identified resorptive defects were then filled with a bioceramic material recently introduced to the Brazilian market, Bio-C Repair (Angelus, Londrina, Brazil). Final obturation was completed with a gutta-percha cone and Bio-C Sealer (Angelus, Londrina, Brazil). After ten months of follow-up, the clinical and radiographic findings were satisfactory. This report illustrates how advances in biomaterials and irrigation protocols may contribute to improved management of resorptive defects, mainly when adequate sealing and biocompatibility are achieved. Orthodontic movement has been described as the second most frequent etiological factor associated with root resorption. As early as 1984, Brady and Lawis documented the occurrence of Pink Tooth of Mummery in a patient undergoing orthodontic treatment. Subsequent reports reinforced this association, including cases described by Nakano et al. (2005) (55), Silveira et al. (2009) (56), and Tunçer et al. (2019) (63). Silveira et al. (2009) (56) presented a double case in which two maxillary central incisors developed pink discoloration at different times following orthodontic therapy. The right central incisor showed signs four months after appliance removal, whereas the left central incisor was affected seven months later. This temporal variation highlights that resorptive processes may manifest months after the initial triggering event, underscoring the importance of continued clinical and radiographic monitoring even after completion of orthodontic treatment. Nakano et al. (2005) (55) and Tunçer et al. (2019) (63) described cases in which orthodontic movement was combined with surgical interventions. In the report by Nakano et al. (2005) the affected tooth was under orthodontic traction in a patient who had previously undergone cleft lip and palate surgery with bone grafting. In the case described by Tunçer et al., a patient with Class II malocclusion underwent an accelerated orthodontic protocol involving mini-implants and piezoincisions. After 27 months, pink discoloration was observed in the maxillary left central incisor, and cone beam computed tomography revealed invasive cervical resorption affecting both central incisors, close to the corticotomy area. These cases demonstrate that when surgical and orthodontic procedures are combined, careful planning and close follow-up are essential to detect potential complications at an early stage. An atypical presentation was described by Gassmann and Arnold (2015) (58), who reported discoloration of the maxillary right central incisor characterized by a darkened hue rather than the classic pink spot. In the absence of a clearly reported traumatic event, the authors attributed the resorption to severe generalized aggressive periodontitis combined with secondary trauma resulting from the buccal positioning of the anterior teeth. Due to the extent of structural damage and the limited feasibility of conservative treatment, extraction was performed, followed by prosthetic rehabilitation. Extraction was likewise unavoidable in the case reported by Korkmaz and Yagci (2017) (60), involving advanced resorption of a maxillary left incisor associated with ectopic eruption and canine transposition. Another unusual situation was described by Korolevsky et al. (2019) (62), in which pink discoloration developed after tooth preparation for a fixed partial denture. Notably, the coronal pulp remained vital, no granulation tissue was clinically evident, and radiographic examination did not reveal typical resorptive defects. The authors suggested that mechanical wear during preparation, thermal effects generated during polymerization of provisional materials, and hyperocclusion induced by the provisional prosthesis may have contributed to the color change. Such atypical manifestations illustrate the diagnostic complexity of Pink Tooth of Mummery and reinforce the need for comprehensive clinical evaluation, detailed history taking, and appropriate imaging to distinguish between true resorptive pathology and other causes of coronal discoloration. In certain circumstances, identifying the etiological factor associated with the pink discoloration is challenging. For this reason, some cases are classified as idiopathic, especially when no history of trauma, orthodontic movement, or other recognized triggering events is reported. Nevertheless, the absence of documented history does not necessarily exclude the possibility of a previous, unnoticed injury. This diagnostic difficulty is evident in the report by Hiremath et al. (2007) (50), in which a patient presented with a pink spot lesion but denied any history of trauma. Although no triggering event was confirmed, irregularities on the incisal edge of the affected tooth suggested prior enamel fractures, raising the possibility of an unrecognized traumatic episode. A similar situation was described by Petel and Fuks (2016) (59) in a two-year-old child with pink discoloration in a deciduous tooth. In this case, the parents did not recall any traumatic event; however, given the child's age and the frequency of minor injuries during early childhood, a traumatic origin could not be ruled out. In contrast, three studies reported cases in which no clear etiological factor could be established despite detailed clinical and radiographic evaluation (61 , 66 , 67). Melo et al. (2017) (61) described a 15-year-old patient with an impacted maxillary left canine presenting a well-defined radiolucent area in the crown. Extraction confirmed pink discoloration during surgery; however, even after thorough investigation, the cause of the resorption remained unidentified. Similarly, Asgary (2023) (66) reported a case of pink discoloration in a maxillary central incisor without any detectable triggering factor, reinforcing that some presentations remain idiopathic despite comprehensive assessment. Adding to this perspective, Kriplani et al. (2024) (67) reported a 28-year-old female patient presenting with pink discoloration of the maxillary central incisor associated with symptomatic apical periodontitis. The patient had a previous history of orthodontic treatment but no history of trauma. Radiographic examination revealed a radiolucent area in the cervical third of the crown compatible with internal resorption, along with periodontal ligament widening. The final diagnosis was Class IV invasive cervical resorption with internal involvement. Management was performed using a non-surgical endodontic approach, with mineral trioxide aggregate (MTA) as repair material and thermoplasticized gutta-percha for obturation, resulting in resolution of symptoms and periapical healing. Although orthodontic treatment was considered a possible contributing factor, no definitive cause was confirmed, further supporting the multifactorial and sometimes uncertain nature of resorptive processes. It is important to differentiate these pathological conditions from physiological root resorption in deciduous teeth, which typically does not alter crown color. However, Ngoc et al. (2019) (51) described an atypical case of a ten-year-old patient presenting pink discoloration in four primary molars, in which internal resorption progressed coronally while the teeth remained clinically stable, leading to visible crown color changes. Although Pink Tooth of Mummery has clear clinical relevance and is frequently associated with internal root resorption, the available evidence remains limited. Most of the studies included in this review consist of isolated case reports or small case series, frequently lacking long-term follow-up and standardized diagnostic criteria. The methodological quality assessment of the included case reports, based on the JBI critical appraisal checklist (Table 2), demonstrated that most studies presented a low risk of bias, with generally consistent reporting of patient characteristics, clinical presentation, diagnostic procedures, and treatment approaches.

Table 2

Nevertheless, some limitations were observed. The domains most frequently affected were the reporting of adverse events and follow-up, which were often unclear or insufficiently described, particularly in older publications. In addition, some studies lacked detailed diagnostic descriptions or clearly defined clinical takeaways. Earlier reports, such as Marshall (1960) (53), showed comparatively higher risk of bias, likely reflecting the diagnostic constraints and reporting standards of the time, whereas more recent studies demonstrated more comprehensive documentation. Overall, despite the predominance of low-risk studies, the variability in reporting specific domains highlights the inherent limitations of case report designs and should be considered when interpreting the findings. Furthermore, in several cases, the etiological factors were inferred from clinical history rather than directly established, introducing potential interpretative bias. Variability in imaging modalities and diagnostic approaches may also compromise the accurate distinction between internal and external resorptive processes. Clinically, these limitations underscore the importance of thorough diagnostic evaluation, including the use of advanced imaging techniques such as cone-beam computed tomography when conventional radiographs are inconclusive. Early identification of subtle signs, such as crown discoloration, may facilitate timely intervention; however, disease progression and treatment outcomes remain unpredictable. In general, the literature demonstrates that Pink Tooth of Mummery may arise in diverse clinical contexts, ranging from clearly identifiable traumatic or iatrogenic factors to situations in which no definitive cause can be established. Its presentation may mimic other resorptive conditions, requiring careful clinical and radiographic evaluation for accurate diagnosis. Variability in progression, prognosis, and therapeutic response further highlights the importance of individualized treatment planning. Clinical awareness of this condition is essential for early recognition and appropriate management.

## Conclusions

Internal root resorption is a multifactorial condition frequently associated with trauma, orthodontic movement, and pulpal alterations. Because it is often asymptomatic, diagnosis depends on careful clinical assessment combined with appropriate imaging, particularly in patients exposed to known risk factors. Cone beam computed tomography has improved diagnostic accuracy, especially in complex cases. When detected early and presenting favorable conditions, conventional endodontic treatment remains the main therapeutic option. Pink Tooth of Mummery represents a clinical manifestation that may be associated with internal root resorption but is not exclusive to it, also occurring in external and, in rare situations, physiological resorption. The literature on this presentation is largely limited to case reports, which restricts the level of available evidence. Further clinical studies are necessary to clarify its etiological mechanisms and to better define treatment protocols.

## Figures and Tables

**Table 1:** Case reports of pink tooth of Mummery, diagnosis, age of patient, tooth affected, and etiologic factor.

Authors	Diagnosis	Age (years)	Tooth affected	Etiological factor
Marshall (1960) [53]		30	21	Trauma
Brady and Lewis (1984) [54]	Internal Root Resorption	34	21	Orthodontic movement
Nakano et al. (2005) [55]	Internal Root Resorption	15	22	Orthodontic movement Cleft lip and palateBone grafting
Hiremath et al. (2007) [50]	Invasive Cervical Resorption	19	11	Idiopathic
Silveira et al. (2009) [56]	Internal Root Resorption	21	11, 21	Orthodontic movement
Segura-Egea et al. (2011) [57]	Internal Root Resorption	29	11	Trauma
Gassmann and Arnold (2015) [58]	Internal Root Resorption	23	11	PeriodontitisSecondary trauma
Petel e Fuks (2016) [59]	Internal Root Resorption	2	51	Idiopathic
Korkmaz and Yagci (2017) [60]	External Root Resorption	13	21	Ectopic eruption
Melo et al. (2017) [61]	Internal Root Resorption	13	23	Idiopathic
Korolevsky et al. (2019) [62]	Internal Root Resorption	67	11	Prosthetic preparation
Ngoc et al. (2019) [51]	Internal Root Resorption	10	54, 64, 74, 84	Physiological
Tunçer et al. (2019) [63]	Invasive Cervical Resorption	16	11, 21	Orthodontic movementPiezoincisions
Deep et al. (2021) [47]	Internal Root Resorption	12	11	Trauma
Munoz-Sanchez et al. (2021) [64]	Invasive Cervical Resorption	10	11, 21	Trauma
Rodrigues et al. (2021) [65]	Invasive Cervical Resorption	28	11	Trauma
Asgary (2023) [66]	Invasive Cervical Resorption	30	21	Idiopathic
Kriplani et al. 2024 [67]	Invasive Cervical Resorption	28	11	Idiopathic

1

**Table 2:** Methodological quality assessment of included case reports using the Joanna Briggs Institute (JBI) critical appraisal checklist. (I): Patient demographics clearly described. (II): Patient history clearly reported. (III): Clinical condition at presentation. IV: Diagnostic methods clearly described. V: Intervention/treatment clearly described. VI: Post-intervention condition reported. VII: Adverse events described. VIII: Follow-up adequately reported. IX: Takeaway lessons provided.

Study	I	II	III	IV	V	VI	VII	VIII	IX	Overall appraisal
Marshall (1960) [53]	Yes	Yes	Yes	Unclear	Yes	Yes	Unclear	Yes	Unclear	Moderate bias
Brady and Lewis (1984) [54]	Yes	Yes	Yes	Yes	Yes	Yes	Unclear	Yes	Unclear	Low bias
Nakano et al. (2005) [55]	Yes	Yes	Yes	Yes	Yes	Yes	No	Unclear	Yes	Low bias
Hiremath et al. (2007) [50]	Yes	Yes	Yes	Yes	Yes	Yes	Unclear	Yes	Yes	Low bias
Silveira et al. (2009) [56]	Yes	Yes	Yes	Yes	Yes	Yes	Unclear	Yes	Yes	Low bias
Segura-Egea et al. (2011) [57]	Yes	Yes	Yes	Yes	Yes	Yes	Yes	Yes	Yes	Low bias
Gassmann and Arnold (2015) [58]	Yes	Yes	Yes	Yes	Yes	Yes	Unclear	Unclear	Yes	Low bias
Petel and Fuks (2016) [59]	Yes	Yes	Yes	Yes	Yes	Yes	Yes	Yes	Yes	Low bias
Korkmaz and Yagci (2017) [60]	Yes	Yes	Yes	Yes	Yes	Yes	Unclear	Yes	Yes	Low bias
Melo et al. (2017) [61]	Yes	Yes	Yes	Yes	Yes	Yes	Unclear	No	Unclear	Moderate bias
Korolevsky et al. (2019) [62]	Yes	Yes	Yes	Yes	Yes	Yes	Yes	Yes	Yes	Low bias
Ngoc et al. (2019) [51]	Yes	Yes	Yes	Yes	Yes	Yes	Yes	Unclear	Yes	Low bias
Tunçer et al. (2019) [63]	Yes	Yes	Yes	Yes	Yes	Yes	Yes	Yes	Yes	Low bias
Deep et al. (2021) [47]	Yes	Yes	Yes	Yes	Yes	Yes	Unclear	Yes	Yes	Low bias
Munoz-Sanchez et al. (2021) [64]	Yes	Yes	Yes	Yes	Yes	Yes	Yes	Yes	Yes	Low bias
Rodrigues et al. (2021) [65]	Yes	Yes	Yes	Yes	Yes	Yes	Yes	Yes	Yes	Low bias
Asgary (2023) [66]	Yes	Yes	Yes	Yes	Yes	Yes	Yes	Yes	No	Low bias
Kriplani et al. 2024 [67]	Yes	Yes	Yes	Yes	Yes	Yes	Yes	Yes	Yes	Low bias

2

## References

[1] Ne RF, Witherspoon DE, Gutmann JL (1999). Tooth Resorption. Quintessence Int.

[2] Patel S, Krastl G, Weiger R, Lambrechts P, Tjäderhane L, Gambarini G, Teng PH (2023). ESE Position Statement on Root Resorption. Int Endod J.

[3] Patel S, Ricucci D, Durak C, Tay F (2010). Internal Root Resorption: A Review. J Endod.

[4] Bell T (1837). The anatomy, physiology, and diseases of the teeth.

[5] Haapasalo M, Endal U (2006). Internal Inflammatory Root Resorption: The Unknown Resorption of the Tooth. Endod Top.

[6] Mummery F (1920). The pathology of” pinksports” on teeth. Br Dent J.

[7] Souza JAS, de Oliveira Alves R, de Toledo PTA, Mota HC, Martins TP, Danelon M, Duque C, Nunes GP (2025). Effect of Disinfection and Tissue Repair with Chloramphenicol-Tetracycline-ZOE Paste on Pulp Therapy of Primary Teeth: A Systematic Review. Clin Oral Investig.

[8] Heboyan A, Avetisyan A, Karobari MI, Marya A, Khurshid Z, Rokaya D (2022). Tooth root resorption: A review. Sci Prog.

[9] Mulumoodi RS, Antony SDP, Adimulapu HS (2020). Association of Age and Gender of Patients with the Type of Tooth Resorption Treated: A Retrospective Analysis. Int J Res Pharm Sci.

[10] J kovljevic A, Kuzmanovic Pficer J, Dragan IF, Knezevic A, Miletic M, Beljic-Ivanovic K, Milasin J, Andric M (2017). The Role of Varicella Zoster Virus in the Development of Periapical Pathoses and Root Resorption: A Systematic Review. J Endod.

[11] Oliveira LCS, Santos DCL, Negrete D, Flaiban E, Bortolin R, Santos RL (2018). Reabsorção Radicular em Tratamento Ortodôntico. Rev Odontol Univ Cid São Paulo.

[12] Ruškytė G, Juozėnaitė D, Kubiliūtė K (2019). Types of Root Resorptions Related to Orthodontic Treatment. Stomatologija.

[13] de Souza BDM, Dutra KL, Reyes-Carmona J, Bortoluzzi EA, Kuntze MM, Teixeira CS, Porporatti AL, De Luca Canto G (2020). Incidence of Root Resorption after Concussion, Subluxation, Lateral Luxation, Intrusion, and Extrusion: A Systematic Review. Clin Oral Investig.

[14] Qassem A, Martins Nda M, da Costa VP, Torriani DD, Pappen FG (2015). Long-Term Clinical and Radiographic Follow-Up of Subluxated and Intruded Maxillary Primary Anterior Teeth. Dent Traumatol.

[15] Kallel I, Douki N, Amaidi S, Ben Amor F (2020). The Incidence of Complications of Dental Trauma and Associated Factors: A Retrospective Study. Int J Dent.

[16] Marasca B, Ndokaj A, Duś-Ilnicka I, Nisii A, Marasca R, Bossù M, Ottolenghi L, Polimeni A (2022). Management of Transverse Root Fractures in Dental Trauma. Dent Med Probl.

[17] Holan G, Yodko E (2017). Radiographic Evidence of Traumatic Injuries to Primary Incisors without Accompanying Clinical Signs. Dent Traumatol.

[18] Abela S, Murtadha L, Bister D, Andiappan M, Kwok J (2019). Survival Probability of Dental Autotransplantation of 366 Teeth over 34 Years within a Hospital Setting in the United Kingdom. Eur J Orthod.

[19] Conway F (2022). Primary Pulpotomies – What Should We Be Using?. Evid Based Dent.

[20] Atasever G, Keceli TI, Uysal S, Gungor HC, Olmez S (2019). Primary Molar Pulpotomies with Different Hemorrhage Control Agents and Base Materials: A Randomized Clinical Trial. Niger J Clin Pract.

[21] Lourenço Neto N, Moretti ABS, Sakai VT, Machado MAAM, Abdo RCC, Oliveira TM (2015). Clinical and Radiographic Outcomes of the Use of Capping Materials in Vital Pulp Therapy of Human Primary Teeth. BDS.

[22] Abiraamasri BL, Gurunathan D (2018). Comparison of Ferric Sulphate and Calcium Hydroxide as a Pulpotomy Agent. Res J Pharm Technol.

[23] Santa KN, Bashar A, Hossain M, Sheikh MAH, Alim MA, Moral AA (2018). Clinical and Radiographic Efficacy of Portland Cement as Pulpotomy Material in Human Primary Molar. Bangladesh Med Res Counc Bull.

[24] Ebrahimi M, Changiz S, Makarem A, Ahrari F (2022). Clinical and Radiographic Effectiveness of Mineral Trioxide Aggregate (MTA) Partial Pulpotomy with Low Power or High Power Diode Laser Irradiation in Deciduous Molars: A Randomized Clinical Trial. Lasers Med Sci.

[25] Jamali Z, Alavi V, Najafpour E, Aminabadi NA, Shirazi S (2018). Randomized Controlled Trial of Pulpotomy in Primary Molars Using MTA and Formocresol Compared to 3Mixtatin: A Novel Biomaterial. J Clin Pediatr Dent.

[26] Haideri S, Koul M, Raj R, Salam SA, Kalim MS, Gupta V (2021). To Evaluate and Compare the Clinical and Radiographic Outcomes of Formocresol, Mineral Trioxide Aggregate, Electrocautery, and Bioactive Glass When Used for Pulpotomy in Human Primary Teeth. J Pharm Bioallied Sci.

[27] Taha NA, Abdulkhader SZ (2018). Full Pulpotomy with Biodentine in Symptomatic Young Permanent Teeth with Carious Exposure. J Endod.

[28] Patel S, Saberi N, Pimental T, Teng PH (2022). Present Status and Future Directions: Root Resorption. Int Endod J.

[29] Abbott PV, Lin S (2022). Tooth Resorption—Part 2: A Clinical Classification. Dent Traumatol.

[30] Aidos H, Diogo P, Santos JM (2018). Root Resorption Classifications: A Narrative Review and a Clinical Aid Proposal for Routine Assessment. Eur Endod J.

[31] Lin S, Moreinos D, Kaufman AY, Abbott PV (2022). Tooth Resorption—Part 1: The Evolvement, Rationales and Controversies of Tooth Resorption. Dent Traumatol.

[32] Andreasen J, Andreasen FM (1994). Textbook and colours atlas of traumatic injuries of the teeth. Copenhagen: Munksgaard.

[33] Wedenberg C, Lindskog S (1987). Evidence for a Resorption Inhibitor in Dentin. Scand J Dent Res.

[34] Soares AJ, Souza GA, Pereira AC, Vargas-Neto J, Zaia AA, Silva EJ (2015). Frequency of Root Resorption Following Trauma to Permanent Teeth. J Oral Sci.

[35] Karthikeson PS, Mahalakshmi J (2021). Assessment of Internal Root Resorption Cases Reported to Private Dental Hospital: A Retrospective Study. Int J Dent Oral Sci.

[36] Nageh M, Ibrahim LA, AbuNaeem FM, Salam E (2022). Management of Internal Inflammatory Root Resorption Using Injectable Platelet-Rich Fibrin Revascularization Technique: A Clinical Study with Cone-Beam Computed Tomography Evaluation. Clin Oral Investig.

[37] Hemmanuer S, Nasim I (2020). Management of Internal Resorption—A Decision Analysis. Int J Pharm Res.

[38] Talpos-Niculescu RM, Nica LM, Popa M, Talpos-Niculescu S, Rusu LC (2021). External Cervical Resorption: Radiological Diagnosis and Literature Review. Exp Ther Med.

[39] Patel S, Mavridou AM, Lambrechts P, Saberi N (2018). External Cervical Resorption—Part 1: Histopathology, Distribution and Presentation. Int Endod J.

[40] Digholkar RS, Aggarwal SD, Kurtarkar PS, Dhatavkar PB, Neil VL, Agarwal DN (2023). Imaging Techniques and Various Treatment Modalities Used in the Management of Internal Root Resorption: A Systematic Review. Endodontology.

[41] Dao V, Mallya SM, Markovic D, Tetradis S, Chugal N (2023). Prevalence and Characteristics of Root Resorption Identified in Cone-Beam Computed Tomography Scans. J Endod.

[42] Martins CM, de Moraes AR, Cruz AJ, Barboza LC, Batista VS, Mori GG, do Prado RL, Matos J, Herrera B, Lacerda PB, Andrada AC (2021). Survey-Based Assessment of Diagnosis through Periapical Radiograph and CBCT and Treatment of Root Resorption with Brazilian and American Dentists and Endodontists. J Clin Exp Dent.

[43] Vidya Bhat S, Sham SB, Musfirat K (2023). Efficacy of 5.25% Sodium Hypochlorite and 810 Diode Laser in Reduction of Microbial Count in Root Canals of Primary Teeth: An In Vivo Study. Res J Pharm Technol.

[44] Sahebalam R, Sarraf A, Jafarzadeh H, Jouybari-Moghaddam M, Seyed-Musavi S (2017). Clinical and Radiographic Success of Electrosurgical Pulpectomy in Primary Teeth. Bull Tokyo Dent Coll.

[45] Estrela C, Decurcio DA, Rossi-Fedele G, Silva JA, Guedes OA (2018). Root Perforations: A Review of Diagnosis, Prognosis and Materials. Braz Oral Res.

[46] Cervino G, Laino L, D’Amico C, Russo D, Nucci L, Amoroso G, Gorassini F, Tepedino M, Terranova A, Gambino D, Mastroieni R, Tözüm MD, Fiorillo L (2020). Mineral Trioxide Aggregate Applications in Endodontics: A Review. Eur J Dent.

[47] Deep A, Thakur S, Singhal P, Chawla D (2021). Management of Root Perforation due to Internal Resorption: A 1-Year Follow-Up Study. Int J Clin Pediatr Dent.

[48] Scott JH (1951). Nasmyth’s membrane. Irish Journal of Medical Science (1926-1967).

[49] Thomas P, Krishna Pillai R, Ramakrishnan BP, Palani J (2014). An Insight into Internal Resorption. ISRN Dent.

[50] Hiremath H, Yakub SS, Metgud S, Bhagwat SV, Kulkarni S (2007). Invasive Cervical Resorption: A Case Report. J Endod.

[51] Ngoc VTN, Son TM, Linh LTT, Anh LQ, Duc NM, Chu DT (2019). An Unusual Tooth Shedding with Internal Resorption: A Case Report. Biomed Hub.

[52] Warnsinck CJ, Shemesh H (2018). Externe Cervicale Wortelresorptie [External Cervical Root Resorption]. Ned Tijdschr Tandheelkd.

[53] Marshall PS (1960). Perforating hyperplasia of the dental pulp: “pink spot”. J R Nav Med Serv.

[54] Brady J, Lewis DH (1984). Internal Resorption Complicating Orthodontic Tooth Movement. Br J Orthod.

[55] Nakano K, Shimizu N, Komura T, Ooshima T (2005). Unusual case of internal resorption in cervical region of maxillary left lateral incisor. Pediatric Dental Journal.

[56] Silveira FF, Nunes E, Soares JA, Ferreira CL, Rotstein I (2009). Double “Pink Tooth” Associated with Extensive Internal Root Resorption after Orthodontic Treatment: A Case Report. Dent Traumatol.

[57] Segura-Egea JJ, Castellanos-Cosano L, Martín-González J, Alonso-Ezpeleta LO, López Frías FJ (2011). Green Discoloration of the Crown after Internal Root Resorption Treatment with Grey Mineral Trioxide Aggregate (MTA). J Clin Exp Dent.

[58] Gassmann G, Arnold WH (2015). Case Report of an Internal Granuloma Investigated by Light and Scanning Electron Microscopy. Head Face Med.

[59] Petel R, Fuks A (2016). Pink Spot: Literature Review and Case Report. J Clin Pediatr Dent.

[60] Korkmaz YN, Yagci F (2017). Multidisciplinary Treatment of Severe Upper Incisor Root Resorption Secondary to Transposed Canine. J Esthet Restor Dent.

[61] Melo NM, Oliveira LJ, Cardoso CAA (2017). Pink Spot with an Internal Resorption: Case Report. J Dent Health Oral Disord Ther.

[62] Korolevsky EV, Komabayashi T, Foran D (2019). Pink Tooth of Mummery in the Maxillary Left Canine after Fixed Partial Denture (FPD) Preparation. Case Rep Dent.

[63] Köseoğlu-Seçgin C, Arman-Özçırpıcı A (2019). An Unusual Case of Invasive Cervical Resorption after Piezosurgery-Assisted En Masse Retraction. Am J Orthod Dentofacial Orthop.

[64] Munoz-Sanchez ML, Decerle N, Devoize L, Nicolas E, Cousson PY, Veyrune JL (2021). Dental Trauma Management in a Young Teenager through Endodontics and Implantology: A Case Report. Healthcare (Basel).

[65] Rodrigues MA, Silva MR, Carvalho AM, Souza CC, Rosas CAP, Cardoso RM, Limoeiro AGS (2021). Invasive Cervical Resorption: Case Report. Res Soc Dev.

[66] Asgary S (2023). Management of Pink Spot Due to Class IV Invasive Cervical Root Resorption Using Vital Pulp Therapy: A Case Report. Iran Endod J.

[67] Kriplani S, Mahapatra J, Sedani S, Ikhar A, Patel A (2024). Clinical Chronicles: A Case Report on Pink Tooth of Mummery. Iran Endod J.

